# Neuronal *Ddit4* Overexpression in the Medial Prefrontal Cortex Reduces Synaptic Density and Impairs Cognitive Function in Mice

**DOI:** 10.1523/ENEURO.0429-25.2026

**Published:** 2026-09-02

**Authors:** Alexander M. Kuhn, Kelly E. Bosis, Madeline M. Mairose, David T. Dadosky, Brandon L. M. Crotchett, Etienne J. Mueller, Kenny Triplett, Lilian Jerow, Lauren Larke Vollmer, Justin L. Bollinger, Eric S. Wohleb

**Affiliations:** ^1^ Department of Pharmacology, Physiology, & Neurobiology, University of Cincinnati College of Medicine, Cincinnati, Ohio 45267; ^2^ Neuroscience Graduate Program, University of Cincinnati College of Medicine, Cincinnati, Ohio 45267; ^3^Molecular & Cellular Biosciences, University of Cincinnati College of Medicine, Cincinnati, Ohio 45267

**Keywords:** behavior, *Ddit4*, dendritic spine, microglia, prefrontal cortex, RNA sequencing

## Abstract

Chronic stress exposure causes neurobiological and behavioral changes that resemble those reported in psychiatric conditions such as major depressive disorder (MDD). Preclinical stress models and studies using postmortem tissue from MDD patients have shown that DNA damage-inducible transcript 4 (*Ddit4*) is increased in the prefrontal cortex (PFC). This is important because DDIT4 negatively regulates the mammalian target of rapamycin (mTOR) pathway, which may lead to behavioral deficits through diminished neuroplasticity and PFC function. Our prior studies indicate that coordinated neuron–microglia interactions contribute to synaptic remodeling in the PFC. The present studies aimed to test the hypothesis that increased neuronal *Ddit4* expression is sufficient to drive structural remodeling of PFC neurons, in part by provoking microglia activation, and this leads to behavioral and cognitive deficits. To this end, we bilaterally infused AAV5-hSyn1-Ddit4-tdTomato or a control vector into the PFC of male Thy1-GFP and C57BL/6 mice and examined molecular, cellular, and behavioral endpoints. Mice with *Ddit4* overexpression (Ddit4-OV) showed no change in passive stress coping, yet exhibited deficits in temporal order memory. Immunohistology analyses showed a decrease in dendritic spine density of Ddit4-OV mice. However, we found no changes in microglia count, microglia size, or nearest neighbor distance. Bulk RNA sequencing of Ddit4-OV PFC revealed increases in transcripts involved with dendrite and synapse function and decreases in transcripts involved with mitochondrial function, implicating mTOR dysregulation. Altogether, these results indicate that *Ddit4* overexpression recapitulates some of the broad molecular, cellular, and behavioral adaptations observed following chronic stress exposure through a cell-autonomous mechanism.

## Significance Statement

This work provides more context for the neurobiological effects of neuronal DNA damage-inducible transcript 4 (*Ddit4*). *Ddit4*, an inhibitor of the mammalian target of rapamycin pathway, exhibits increased expression in the prefrontal cortex (PFC) of both rats exposed to preclinical chronic stress models and humans diagnosed with major depressive disorder. Our findings demonstrate that *Ddit4* overexpression specifically in neurons is sufficient to reduce spine density in the PFC, impair temporal order memory, and induce transcriptional changes associated with stress and depression. These results indicate that neuronal *Ddit4* can disrupt PFC function and cognitive performance in a cell-autonomous manner.

## Introduction

The incidence rate of depression and anxiety disorders continues to rise worldwide, and this creates a substantial socioeconomic burden (Goodwin et al., 2022; Kavelaars et al., 2023). Chronic stress is often implicated in the etiology of these psychiatric disorders as adverse life events are a significant risk factor in developing major depressive disorder (MDD), post-traumatic stress disorder (PTSD), and other related disorders (Maeng and Milad, 2017; Monroe and Harkness, 2022). To this end, preclinical chronic stress models can be utilized to better understand the neurobiological adaptations that contribute to behavioral and cognitive impairments. Notably, accumulating evidence shows that chronic stress exposure in rodents decreases synapse density and function in the PFC (Radley et al., 2006; Kang et al., 2012; Qiao et al., 2016), and this contributes to deficits in temporal order memory (Yuen et al., 2012; Nikolin et al., 2021). Glucocorticoids have been shown to be a key component of stress-induced changes in the brain (Wellman, 2001; Liston and Gan, 2011). Upon binding glucocorticoid receptors (GR) within the cytosol, GR can bind to glucocorticoid response elements (GREs) in the genome and modulate gene transcription (Koning et al., 2019). One such gene that has been demonstrated to be directly induced by GR activation is DNA damage-inducible transcript 4 (*Ddit4*; Wolff et al., 2014).

*Ddit4*, also known as *Redd1* or *RTP801*, is a stress-responsive protein that regulates metabolism, inflammation, and cellular homeostasis. As its name implies, *Ddit4* expression is observed in conditions that cause cellular stress or DNA damage (Shoshani et al., 2002; Whitney et al., 2009; Hayasaka et al., 2014; Kim et al., 2023). Interestingly, patients diagnosed with MDD and rats exposed to chronic stress have shown elevated *Ddit4* expression in the PFC (Ota et al., 2014). This is relevant because *Ddit4* is known to inhibit the mammalian target of rapamycin (mTOR) pathway (Brugarolas et al., 2004), which regulates neural growth (Lu et al., 2014), synaptic plasticity (Tang et al., 2002), and cognitive performance (Graber et al., 2013). Additionally, chronic stress exposure leads to alterations in the mTOR pathway in the PFC (Zhang et al., 2023). This suggests that *Ddit4* contributes to the neurobiological and behavioral changes observed in both stress and depression. Indeed, one study has shown that modulation of *Ddit4* through genetic knock-out or AAV-mediated overexpression in rat PFC was able to ameliorate or exacerbate stress effects, respectively. These prior studies showed that behavioral changes, such as anhedonia, passive stress coping, and reduced exploration, coincided with deficits in neuronal dendritic spine density (Ota et al., 2014). Of note, this work used global genetic knock-out and viral-mediated overexpression with the broad immediate/early promoter enhancer of cytomegalovirus. With *Ddit4* being expressed in multiple cell types (Prabakaran, 2015; Tirado-Hurtado et al., 2018; Sadick et al., 2022), we sought to further understand the effects of *Ddit4* expression in a cell type–specific approach.

Recent studies demonstrate that non-neuronal cells, particularly microglia, contribute to the neurobiology of stress (Yirmiya et al., 2015; Wohleb et al., 2016; Bollinger and Wohleb, 2019). Under chronic stress conditions, microglia exhibit alterations in density, morphology, and molecular signature. Notably, multiple studies have shown an increase in microglial phagocytosis of dendritic material following chronic stress exposure (Milior et al., 2016; Horchar and Wohleb, 2019). More so, evidence exists that chronic stress stimulates release of neuron-derived factors via GR signaling, such as colony-stimulating factor 1 (*CSF1*), which directly modulates microglia function (Wohleb et al., 2018). It is not yet clear if increased *Ddit4* in neurons leads to comparable neuron–microglia interactions and the extent to which neuronal *Ddit4* overexpression is sufficient to recapitulate synaptic and behavioral deficits observed after chronic stress exposure. Therefore, in this study, we sought to investigate whether *Ddit4* overexpression in pyramidal neurons within the PFC of mice is sufficient to phenocopy the molecular, neurobiological, and behavioral changes observed with stress exposure.

## Materials and Methods

### Animals

Male Thy1-GFP(M) and C57BL/6J mice were obtained from Jackson Laboratory [Tg(Thy1-EGFP)MJrs/J; #007788, C57BL/6J; #000664]. Studies were performed with mice 6–10 weeks old. Mice were group-housed (four/cage) in 11.5″ × 7.5″ × 6″ polypropylene cages under a 14–10 h light/dark cycle with *ad libitum* access to water and rodent chow. Animal use and procedures were in accordance with the National Institutes of Health guidelines and approved by the University of Cincinnati Institutional Animal Care and Use Committee.

### Stereotactic surgery

Mice were anesthetized with ketamine:xylazine (80:10 mg/kg) followed by stereotactic surgery. A control (AAV5-Syn1-TdT) or *Ddit4*-overexpression (AAV5-Syn1-Ddit4-TdT) virus was bilaterally infused (0.5 µl; 0.1 µl/min) into the PFC (AP, +2.0 mm; ML, ±0.2 mm; DV, −2.8 mm). Both viruses were ordered from VectorBuilder [catalog #AAV5MP(VB220327-1098qcf)-K0]. Syringes were left in place for 5 min after infusion to limit viral spreading in the needle tract. Mice received daily injections of carprofen (5 mg/kg, i.p.) over 3 d for pain relief. Mice were handled intermittently over 4 weeks to allow for surgical recovery and viral infection/expression.

### Behavior and cognitive testing

Temporal object recognition (TOR) and forced swim test (FST) were conducted on separate days as previously described (Bollinger et al., 2023). All behavioral tests were performed in a normally lit room (white light), between 0800 and 1000 h. Mice were allowed to habituate to this room for 30 min prior to testing. In the first trial of the TOR (Trial 1), mice were placed in a plastic arena with two plastic Lego trees secured to the bottom of the arena. After a 30 min latency, mice were placed in the same arena with Lego blocks (Trial 2). An hour after the second trial, mice were placed in the arena with one block and one tree, counterbalanced to prevent bias (Test). The time spent exploring each of these objects was measured, and a discrimination index (DI) was calculated as follows:

$$\mathrm{DI}=\;\frac{t_{1}-t_{2}}{\left(t_{1}+t_{2}\right)}.$$

In the above equation, DI is the discrimination index, *t*_1_ is the time spent during the test phase exploring the object from the first trial, and *t*_2_ is the time spent during the test phase exploring the object from the second trial. For each trial of the TOR, mice were given 5 min to explore the arena and objects. Time spent exploring an object was defined as sniffing, biting, or rubbing against the object. For the FST, mice were placed in a 2 L beaker of water (24° ± 1°C) for 10 min, and immobility was measured between minutes 2 and 6.

### RNA isolation and real-time quantitative PCR

RNA was extracted from whole brain regions (PFC) using TRIzol reagent according to the manufacturer's protocol (Invitrogen). Samples were reverse transcribed, and real-time quantitative PCR was conducted as previously described (Wohleb et al., 2018; Horchar and Wohleb, 2019).

### Immunohistology

Mice were transcardially perfused with PBS followed by 4% paraformaldehyde (PFA) ∼24 h after behavioral assays. Brains were postfixed in 4% PFA for 24 h and incubated in 30% sucrose until cryoprotected. Brains were then rapidly frozen and sectioned on a Leica CM2050 S cryostat (40 µm). Free-floating sections containing the PFC were selected for analysis. Sections were washed, blocked in 1% bovine serum albumin (Thermo Fisher Scientific; #BP9703) with 5% normal donkey serum (EMD Milipore; #S30-100ML) for 1 h at room temperature, washed, and then incubated with primary antibody: rabbit anti-IBA1 (1:1,000, Wako; 019-19741) overnight at 4°C. Sections were then washed and incubated with conjugated secondary antibody overnight at 4°C (1:1,000, Invitrogen; Alexa Fluor 488; Alexa Fluor 647). Sections were subsequently washed, mounted on gel-coated slides, and coverslipped with Fluoromount-G (Thermo Fisher Scientific; 00-4958-02).

### Quantitative immunofluorescence

Confocal images were captured from adjacent brain sections in both hemispheres of the mPFC (3–4 sections/sample spanning the rostral–caudal axis, 2.10–1.34 mm bregma; Nikon C2+ microscope). For analysis of IBA1+ material, tissue sections were imaged using a 20× objective (NA, 0.95; *z*-stack, 0.8 µm; image size, 1,024 × 1,024). Individual IBA1+ cell counts were obtained by hand, and cell density was calculated (cells per mm^2^). To determine microglial area, a threshold was applied to images, the total area was recorded (µm^2^), and area per microglial cell was calculated (µm^2^/cell). To examine dendritic spine density, images from layer I of the PFC were captured using a 40× objective (NA, 0.95; zoom, 2×; *z*-stack, 0.2 µm; image size, 1,024 × 1,024). Spines were counted using Imaris 10. Average spine densities were calculated for each mouse based on 2–5 dendritic segments. Spines were classified using the MATLAB-based “Classify Spines Xtension” in Imaris 10. “Stubby” spines were defined as spines exhibiting a total length <1 µm. “Mushroom” spines were defined as spines exhibiting a total length <3 µm and a maximum head width >0.7 µm. “Long Thin” spines were defined as spines that didn't fit the criteria for either “Stubby” or “Mushroom” spines. Spine heads were defined as the distal 25% of the total spine.

### RNA sequencing

Total RNA was extracted from select samples based on initial PCR analyses. Directional polyA RNA-seq were performed by the Genomics, Epigenomics and Sequencing Core at the University of Cincinnati (Qiu et al., 2023). Briefly, the quality of total RNA was QC analyzed by Bioanalyzer (Agilent), which showed high quality (RIN score >9). To enrich polyA RNA for library preparation, the NEBNext Poly(A) mRNA Magnetic Isolation Module (New England BioLabs) combined with the SMARTer Apollo automated NGS library prep system (Takara Bio) was used with 1 µg of total RNA as input. Next, an NEBNext Ultra II Directional RNA Library Prep kit (New England BioLabs) was used for library preparation under a PCR cycle number of eight. After library QC via Bioanalyzer and quantification via real-time qPCR (NEBNext Library Quant Kit, New England BioLabs), individually indexed libraries were proportionally pooled and sequenced using the NextSeq 550 sequencer (Illumina). Under the sequencing setting of single read 1 × 85 bp, ∼25 M reads per sample were generated. Fastq files for downstream data analysis were then automatically generated via the Illumina BaseSpace Sequence Hub after sequencing. To identify differentially expressed genes (DEGs), bioinformatic analysis was performed via BaseSpace app RNA-Seq Alignment v2.0.2 (option of mark duplicates, unchecked; Parekh et al., 2016) followed by RNA-Seq Differential Expression app version 1.0.1. Specifically, reference genome *Mus musculus*/MM10 (RefSeq) was used for reads alignment, which was performed under a first strand setting. The analysis used STAR for alignment and Salmon for quantification and assigned Transcripts Per Million to genes and transcripts. The alignment result was then seamlessly used as input in the RNA-Seq Differential Expression app, which performed the analysis via DESeq2 to generate reports for the differential gene expression, dispersion plots, and differential expression heatmap (Extended Data Fig. 3-1*A*–*C*). A volcano plot was generated using the web app VolcaNoseR (Goedhart and Luijsterburg, 2020; Fig. 3*A*). Within the DESeq2 analysis, average gene counts were calculated for each respective group (Con and Ddit4-OV) to find differences in expression (log_2_ fold change). Of all the genes (24,972), a filter was applied to remove genes with low counts, leaving 9,389 genes. A Wald test was then performed on these genes and genes with a *q* value <0.05 were determined to be DEGs. All data will be made publicly available at the Gene Expression Omnibus (GEO) and a link will be provided in the article text upon acceptance.

### Pathway enrichment analysis

Pathway enrichment analysis (PEA) was done using ShinyGO 0.85.1 (Ge et al., 2020). The pathways considered for this analysis were from three subsets of the Gene Ontology (GO) database: GO: Molecular Function, GO: Cellular Component, and GO: Biological Process. ShinyGO uses the hypergeometric test and false discovery rates (FDRs) are computed via the Benjamini–Hochberg method to correct for multiple testing. To filter pathways, those containing >100 genes were considered from the GO: Molecular Function and GO: Cellular Component subsets, and pathways containing >200 genes were considered from the GO: Biological Process subset. Pathways were included with FDR < 0.01 and listed based on the average of their rank based on FDR and Gene Enrichment score.

### Upstream regulator analysis

Upstream regulator analysis (URA) was conducted using the QIAGEN Ingenuity Pathway Analysis (IPA) software on the DEGs identified from RNA sequencing. Upstream regulators were considered “activated” if they exhibited a *p* value of overlap <0.05 and an activation *z*-score >2. Upstream regulators were considered “inhibited” if they exhibited a *p* value of overlap <0.05 and an activation *z*-score less than −2. In the analysis software, various molecule types were detected such as transcriptional regulators, enzymes, kinases, ion channels, etc. Transcriptional regulators were of most relevance and interest in this study, so they were singled out.

### Experimental design and statistical analysis

This study was designed to make direct comparisons between two groups (Control vs Ddit4 overexpression). Data were analyzed using GraphPad Prism 10.4.1. Significance value (*α*) was established as 0.05. For every graph with two groups, differences between group means were evaluated using a two-tailed Student's *t* test. For the analysis of TOR exploration time, a two-way repeated-measures ANOVA was used. For the analysis of spine subtype densities, a standard two-way ANOVA was used. Multiple comparisons within ANOVAs were calculated using Šídák's multiple-comparisons test. Outliers were detected using the Grubbs' test.

## Results

### Neuronal Ddit4 overexpression in the PFC impaired temporal order memory

**Figure 1.**
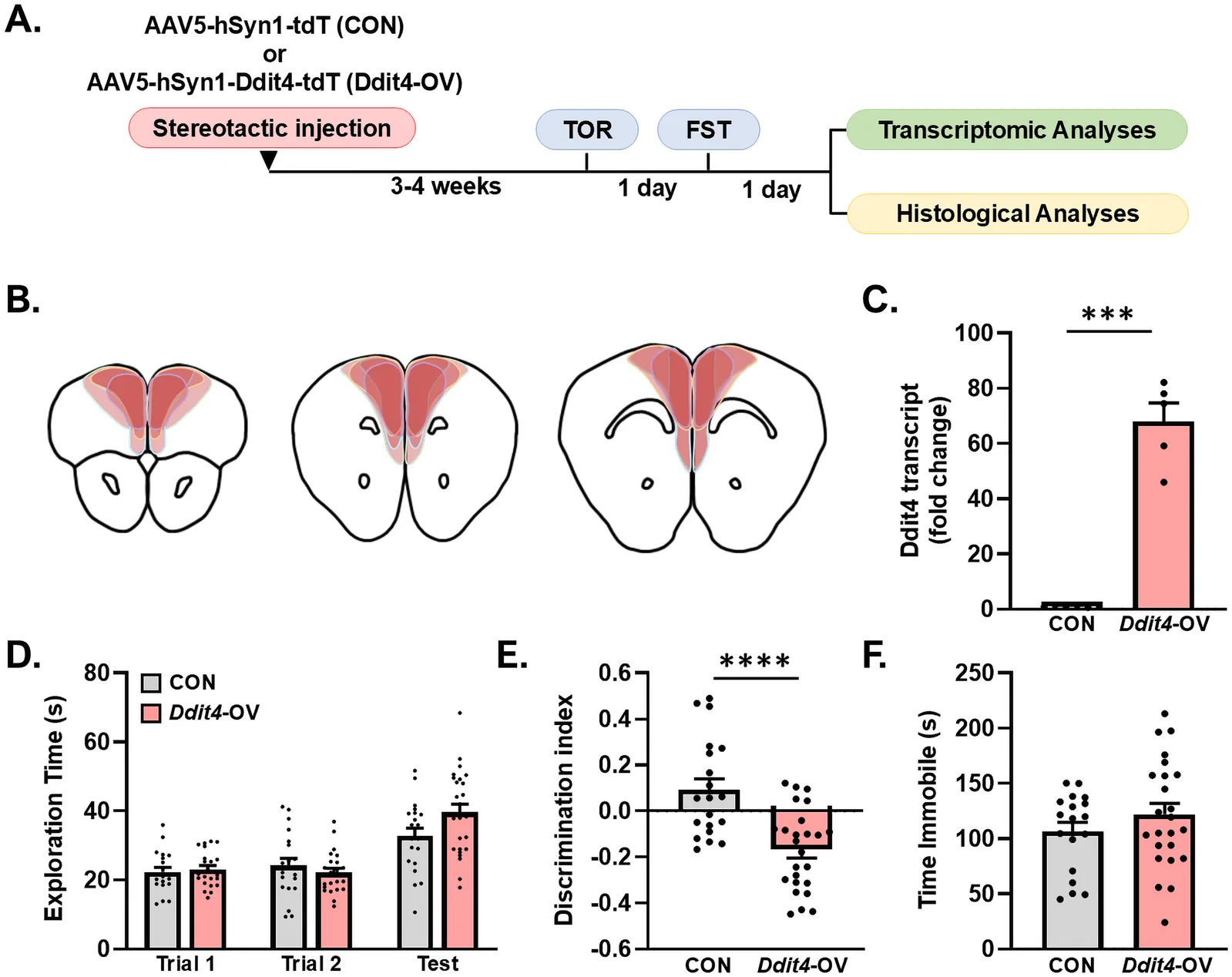
Neuronal Ddit4 overexpression in the PFC impaired temporal order memory. ***A***, Male Thy1-GFP(M) and C57BL/6J mice were injected with either AAV5-Syn1-tdT (Con) or AAV5-Syn1-tdT-Ddit4 (Ddit4-OV) in the PFC. All mice were exposed to two behavioral assays, the TOR test and then the FST. Mice were then either dedicated to histological analyses or transcriptomic analyses. ***B***, Viral spread was confirmed in multiple regions of the PFC. This was illustrated through tracing of transfected cell bodies. ***C***, Fold change of Ddit4 transcripts following real-time quantitative PCR (RT-qPCR; *n* = 6/group, one outlier removed based on Grubbs' test). ***D***, Exploration time or total time interacting with objects, for each trial of the TOR behavioral assay (*n* = 20–25). ***E***, DI in the TOR test (*n* = 20–25/group). ***F***, Immobility time between minutes 2 and 6 in the FST (*n* = 18–24/group). Error bars indicate mean ± SEM. Graphed points indicate data derived from individual animals. *p < 0.05, ***p < 0.001, ****p < 0.0001 for two-tailed Student's *t* test or Wald's test.

To understand the neurobiological and behavioral effects of neuronal *Ddit4* overexpression in the PFC, male mice were given a bilateral PFC infusion of a control vector (CON) or a *Ddit4*-containing viral vector (*Ddit4*-OV; Fig. 1*A*). After 3–4 weeks of recovery, mice were weighed and put through two behavioral assays: the TOR test and the FST on separate days. These tests were chosen as they've been shown to measure behaviors regulated by the PFC. Specifically, FST measures passive-stress coping and TOR measures temporal order memory. After collecting behavioral data, one subset of mice was perfused, and brains were collected for immunohistology to confirm the spread of our viral injections into the PFC. Both AAV constructs expressed tdTomato (tdT), a red fluorescent protein, which was used as a visual marker for viral spread. Figure 1*B* shows the relative distribution of tdT+ cell bodies from representative brain sections across the PFC (bregma 2.22–1.25 mm). In a separate cohort, brains were dissected for initial gene expression analyses in the PFC. RT-PCR analysis revealed that the neuron-specific *Ddit4* overexpression viral vector caused an average 68-fold increase in *Ddit4* transcript in the PFC (*t* = 10.99; df = 9; *p* < 0.0001; Fig. 1*C*). In these studies, we did not observe any weight change over 2 weeks following stereotaxic injection (data not shown).

In the TOR behavioral assay, four measurements were recorded: total exploration or engagement time in each phase (Trial 1, Trial 2, and Test) and a DI. Repeated-measures ANOVA showed a main effect of trial (*F*_(1.567,62.69)_ = 36.81; *p* < 0.0001), but there was no effect of AAV construct on total exploration time (Fig. 1*D*). Additionally, multiple comparisons revealed *Ddit4*-OV mice exhibited no differences in exploration time in each trial of the TOR behavioral assay in comparison with control mice. However, *Ddit4*-OV mice showed a significant decrease in the DI compared with controls (*t* = 4.49; df = 43; *p* < 0.0001; Fig. 1*E*). In the FST behavioral assay, there were no changes in time immobile as a result of *Ddit4* overexpression (Fig. 1*F*).

### Ddit4 overexpression in PFC neurons reduces dendritic spine density but did not affect microglial morphology

**Figure 2.**
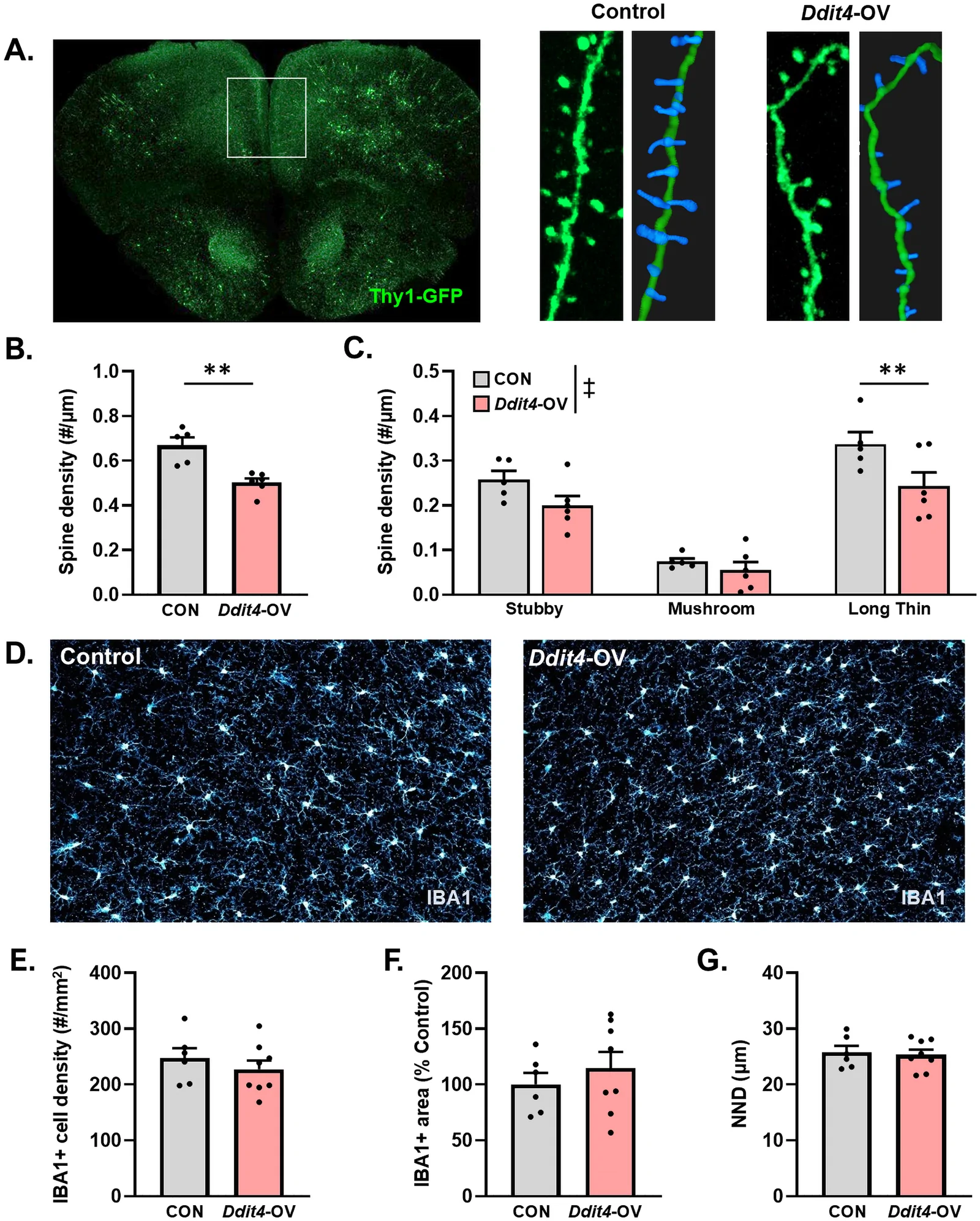
Ddit4 overexpression in PFC neurons reduces dendritic spine density but did not affect microglial morphology. ***A***, Schematic showing the region of the PFC imaged (left). Representative 40× confocal images of apical dendrites in layer I of the PFC and their corresponding reconstruction using the Imaris software (right). ***B***, Average apical dendritic spine density of dendrites in layer I of the PFC (*n* = 5–6/group). ***C***, Apical dendritic spine density of three morphologically based spine classes (*n* = 5–6/group). ***D***, Representative 20× confocal images of IBA1-labeled microglia in layers I–II of the PFC. ***E***, Average microglia count per millimeter square in the PFC (*n* = 6–8/group). ***F***, Average IBA1^+^ cell size as a percentage of the control cohort cell size (*n* = 6–8/group). ***G***, Average NND between microglia in layers I–II of the PFC (*n* = 6–8/group). Error bars indicate mean ± SEM. Each data point represents the average from one mouse. Graphed points indicate data derived from individual animals. *p < 0.05; **p < 0.01; ****p < 0.0001 for two-tailed Student's *t* test or Wald's test.

Many previous studies have established that neurons and microglia in the PFC undergo morphological changes following chronic stress exposure (McEwen et al., 2016; Kuhn et al., 2024). To this end, histological analyses were pursued to observe the effect of neuronal *Ddit4* overexpression on morphological features of pyramidal neurons and microglia within the PFC. Thy1-GFP mice were utilized due to their endogenous green fluorescent protein (GFP) within neurons (Feng et al., 2000). Three-dimensional reconstructions of apical dendrites in layer I of the PFC were rendered from *z*-stacks of confocal images using the Imaris software (Fig. 2*A*). Consistent with prior reports, *Ddit4*-OV mice showed a significant reduction in dendritic spine density compared with controls (*t* = 4.37; df = 9; *p* < 0.002; Fig. 2*B*). To determine if specific subtypes were affected, dendritic spines were classified as stubby, mushroom, or long-thin based on established parameters as listed in the methods. These analyses revealed a main effect of viral construct (*F*_(1,27)_ = 9.636; *p* = 0.004) across dendritic spine subtypes as well as a main effect of spine classification (*F*_(2,27)_ = 52.88; *p* < 0.0001). Post hoc comparisons revealed that *Ddit4*-OV significantly reduced the density of long thin dendrites (*t* = 2.94; df = 27; *p* < 0.007) and caused a nonsignificant decrease in the density of stubby dendrites (*p* < 0.08; Fig. 2*C*).

Additional immunohistology used the ionized calcium-binding adapter molecule 1 (IBA1) antibody to assess the density and morphology of microglia (Imai et al., 1996; Jurga et al., 2020). Data were generated from max-intensity confocal images of IBA1^+^ fluorescence across multiple sections of the PFC (Fig. 2*D*). No differences in microglial cell density (Fig. 2*E*), normalized area per cell (Fig. 2*F*), and nearest neighbor distance (NND; Fig. 2*G*) were observed in *Ddit4*-OV mice compared with controls.

### Neuronal Ddit4 overexpression decreased transcripts involved with cellular energetics and translation and increased transcripts involved with synaptic function and behavior

**Figure 3.**
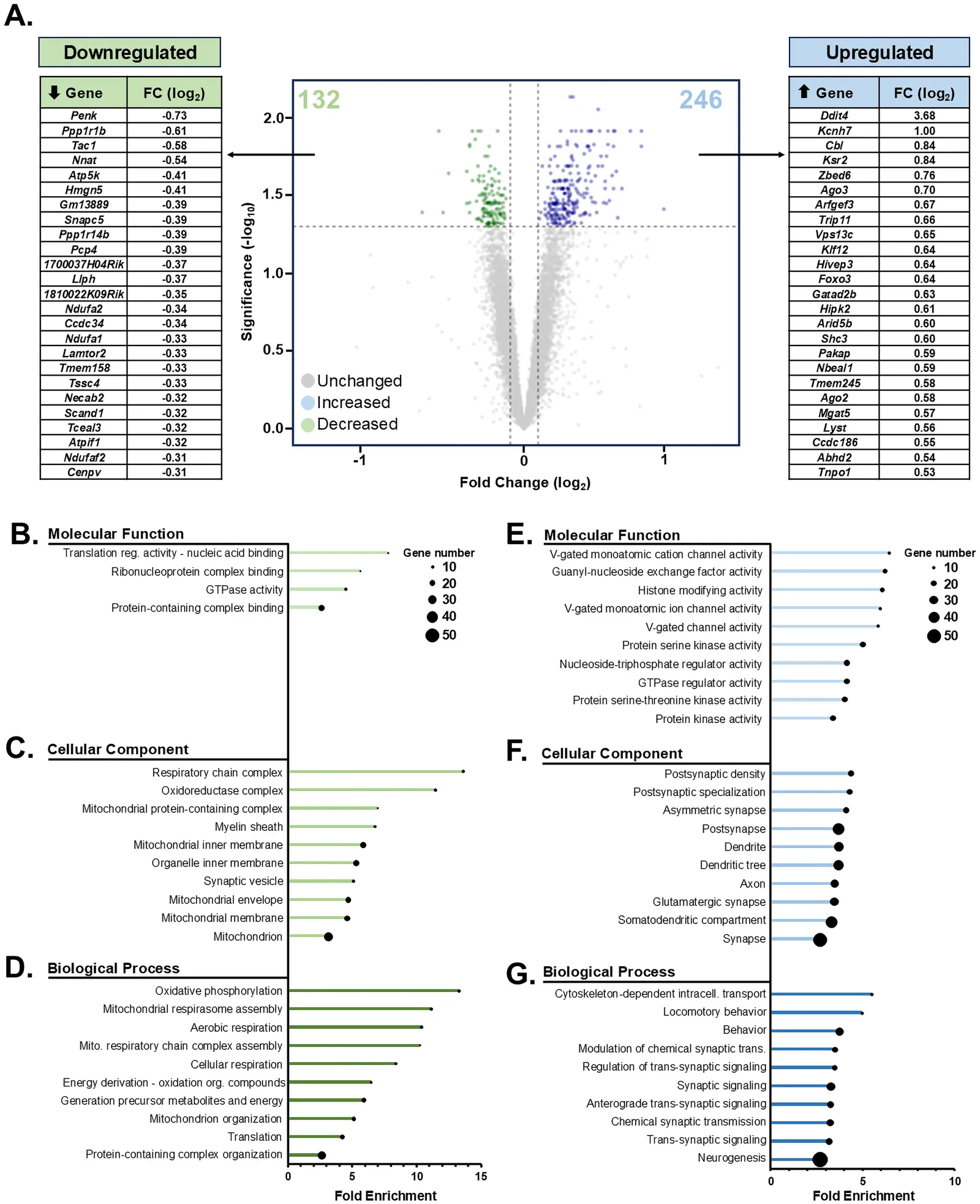
Neuronal Ddit4 overexpression decreased transcripts involved with cellular energetics and translation and increased transcripts involved with synaptic function and behavior. (***A***), Volcano plot of genes. DEGs were defined as genes with a *p*-value <0.05 and a fold change with an absolute value >0.1. The 246 blue dots represent genes that exhibited an increase in expression. The 132 green dots represent genes that exhibited a decrease in expression. (*n* = 6/group). Top 25 down- and upregulated genes based on fold change are displayed along with individual fold change (FC; log_2_) values to the left and right, respectively. ***B–D***, ShinyGO analysis of downregulated DEGs. Pathways with FDR < 0.01 were included and ranked based on average of FDR and Gene Enrichment score. Size of black dots represent the number of DEGs identified in GO pathways. Top 10 GO Molecular Function (***B***), GO Cellular Component (***C***), and GO Biological Process (***D***) pathways from PEA of downregulated genes. ***E–G***, ShinyGO analysis of upregulated DEGs. Top 10 GO Molecular Function (***E***), Cellular Component (***F***), and Biological Process (***G***) pathways from PEA of upregulated genes. More information on RNA sequencing can be found in the extended data figure (Extended Data Fig. 3-1).

To gain insight into the transcriptional changes underlying the neurobiological and behavioral effects of *Ddit4* overexpression, bulk RNA was isolated from dissected PFC samples (*n* = 6/group), sequenced, and used to generate raw gene counts. Using a DESeq2 analysis, 378 genes were determined to be differentially expressed between the control and *Ddit4*-OV groups (Extended Data Fig. 3-1*D*). Of the 378 DEGs, 132 transcripts had decreased expression, and 246 transcripts had increased expression and in the *Ddit4*-OV group compared with the control group (Fig. 3*A*).

10.1523/ENEURO.0429-25.2026.f3-1 Figure 3-1 (A) Scatterplot of log fold change and normalized count for each gene. Red dots represent differentially expressed genes (DEGs). (B) Plot of dispersion estimates for each gene. (C) Differential expression heatmap for each sample. (D) Schematic summary of RNA sequencing process. Download Figure 3-1, TIF file.

Of the 132 downregulated genes, the top 25 based on fold change are shown in Figure 3*A*. All 132 downregulated genes were entered into ShinyGO to conduct PEA. For the PEA, three aspects of the Gene Ontology databases were considered: Molecular Function, Cellular Component, and Biological Process. PEA of the downregulated genes revealed significant overlap with multiple pathways within GO gene sets. The top pathways in “Molecular Function” were gene clusters involved with transcription and translation (Fig. 3*B*). The pathways noted in “Cellular Component” were related to metabolic processes, mitochondrial function, and synaptic transmission (Fig. 3*C*). Finally, the pathways in “Biological Process” included gene clusters related to mitochondrial function, cellular respiration, and translation (Fig. 3*D*).

Of the 246 upregulated genes, the top 25 based on fold change were pulled out and displayed in a table (Fig. 3*A*). All 246 upregulated genes were entered into ShinyGO to conduct PEA. In a similar fashion to the analysis of the downregulated genes, the Molecular Function, Cellular Component, and Biological Process aspects of the GO database were considered. PEA of the upregulated genes revealed significant overlap with multiple pathways within GO gene sets. The top pathways in “Molecular Function” were related to ion channels, histone modifications, and cell signaling (Fig. 3*E*). The pathways included in “Cellular Component” were related to dendritic architecture and postsynaptic density and transmission (Fig. 3*F*). Finally, the top pathways under “Biological Process” had gene clusters related to behavior, synaptic signaling, and neurogenesis (Fig. 3*G*).

### Upstream regulator analysis reveals activation state of several pathways

To identify potential upstream regulators of the transcriptomic changes caused by *Ddit4* overexpression, URA was performed on the list of DEGs found in the RNA sequencing analysis. Seven upstream transcriptional regulators were identified with three predicted to be “activated” (i.e., activation *z*-scores >2 and *p* values <0.05) and four predicted to be “inhibited” (i.e., activation *z*-scores <2 and *p* values <0.05). The three “activated” transcriptional regulators were ZHX2, EIF6, and IGF2BP1. The four “inhibited” transcriptional regulators were TEAD1, TOX, CREB1, and SIRT1.

## Discussion

Since its discovery in 2002, the stress-sensitive *Ddit4* gene has captured the interest of many biological researchers studying various tissues, physiological states, and disease models (Tirado-Hurtado et al., 2018). This remains true in the field of neuroscience, as studies have demonstrated that the absence or overexpression of *Ddit4* mediates stress effects on animal behavior and neuronal structure in the PFC (Ota et al., 2014). Our studies sought to expand upon this by assessing the behavioral effects of neuron-specific *Ddit4* overexpression in the PFC and the extent to which this engages neuroimmune pathways or other molecular adaptations.

Our behavioral analyses reveal that neuronal *Ddit4* overexpression in the PFC impaired performance in TOR, which can be interpreted as a deficit in temporal order memory or object recency memory. Notably, the diminished TOR performance is similar to deficits observed in prior studies using CUS exposure (Horchar and Wohleb, 2019; Woodburn et al., 2021). These results suggest that stress-induced temporal order memory deficits are mediated, in part, by increases in *Ddit4* expression. Our findings are in line with other studies that report increased expression of *Ddit4* is associated with memory impairments and that *Ddit4* knockdown prevented memory deficits in an Alzheimer's disease model (Yi et al., 2020). Intriguingly, we failed to replicate the reduction in passive-stress coping following *Ddit4* overexpression shown in Ota et al. Possible explanations for these contrasting results could be differences in preclinical animal models (rats vs mice), viral constructs, and experimental timelines.

Another important finding was that neuronal *Ddit4* overexpression in the PFC led to a reduction in dendritic spine density on apical dendrites of pyramidal neurons (Fig. 2*A*,*B*). The observed deficit in dendritic spine density is reminiscent of those reported in several chronic stress models (Radley et al., 2006; Wohleb et al., 2016; Woo et al., 2021). Moreover, subtype analyses revealed significant decreases in long, thin spine density, but not in stubby or mushroom spines (Fig. 2*C*). This observation is consistent with stress studies that have shown spine subtype-specific plasticity following chronic variable stress exposure (Radley et al., 2013). Long, thin spines are considered to be immature synapses that are more vulnerable to remodeling than larger, more stable mushroom spines. Therefore, it has been hypothesized that long, thin spines are important for adaptive learning and memory (Sebastian et al., 2013). The results from our experiments suggest that neuronal *Ddit4* overexpression negatively affects density of long, thin spines in the PFC, which may have contributed to the impairment of object recency memory observed in the TOR assay.

Contrary to our initial hypothesis, histological analyses of microglia indicate that neuronal *Ddit4* overexpression did not have an overt effect on microglia morphology. Indeed, we found no effect on microglia counts, average cell size, and NND (Fig. 2*D*–*G*). In the field, it is understood that microglia morphology is not indicative of phenotype or function, yet it does provide relevant measures of microglial responses to various challenges (Henze et al., 2026). Multiple studies have indicated that chronic stress exposure can change microglial density and morphology (Tynan et al., 2010; Sequeira and Bolton, 2023; Vu et al., 2023), but a consensus in the field about whether density increases or decreases has not yet been established. In our case, we found no change in these microglial parameters following neuronal *Ddit4* overexpression (Fig. 2*D*–*G*). However, we still observed a decrease in spine density (Fig. 2*B*,*C*), which suggests that *Ddit4* overexpression leads to synaptic spine deficits in a cell-autonomous manner, independent of microglial involvement. As DDIT4 is known to be a negative regulator of mTOR, it is possible that this occurs through dysregulation of mTOR activity, though more studies would be needed to confirm this mechanism.

Through RNA sequencing and pathway enrichment analysis, we show that neuronal *Ddit4* overexpression increased transcripts involved with regulating synapse function, including synaptic scaffolding proteins (*Shank1*, *Shank2*, *Homer2*, *Bsn*), neurotransmitter receptor subunits (*Gabrb1*, *Chrm3*), and ion channels (*Cacna1b*, *Kcna2*, *Kcnb1*, *Scn1a*; Fig. 3*E*). This is somewhat counterintuitive to the observed decrease in dendritic spine density (Fig. 2*B*). It is possible that these gene expression changes are compensatory and related to ongoing structural remodeling of neurons in the PFC. It is also possible that this discordance can be attributed to impaired mTORC1 signaling and protein translation. When active, mTORC1 can initiate protein translation (Garza-Lombó and Gonsebatt, 2016), and mTOR inhibitors decrease expression of genes regulating translation (Larsson et al., 2012). If *Ddit4* overexpression effectively inhibited mTORC1 and impaired translation, this may lead to a buildup of transcripts. Additional studies examining the phosphorylation state of mTORC1 as well as eukaryotic initiation factor 4E-binding protein (4E-BP1) and S6 kinase (S6K1; Garza-Lombó and Gonsebatt, 2016) would be needed to verify these speculations.

RNA sequencing and pathway enrichment analysis also detected decreases in transcripts involved with mitochondrial components and oxidative phosphorylation. This finding is intriguing because mitochondrial dysfunction has been established as a key part of the neuronal atrophy hypothesis of depression and this dysfunction is routinely observed in neurons following chronic stress exposure (Woo et al., 2021; Krasner et al., 2025). We also know that DDIT4 is a negative regulator of mTOR. Given the ability of mTORC1 to promote transcription of mitochondrial proteins (Morita et al., 2013), change the ATP-producing capacity of mitochondria (Schieke et al., 2006), and alter mitochondria morphology (Morita et al., 2017), this along with our observations suggests that increases in neuronal *Ddit4* could affect mitochondrial function. Indeed, some of the genes associated with mitochondria function found to be downregulated after *Ddit4* overexpression, such as *Rab3a*, *Nme2*, and *Ppp1ca*, can affect neuron growth and synaptic plasticity (Hamasaki et al., 2016; Foley et al., 2021; Yang et al., 2023). Together, this evidence suggests that the effect of *Ddit4* overexpression on mitochondrial function may play a part in the decrease in spine density that we observed.

Of all the predictions from the Upstream Regulator Analysis, the predicted “inhibited” state of CREB1 stands out due to its established role as a molecular regulator of plasticity (Wolf et al., 2016) and its ability to activate mTOR signaling (Kudalkar et al., 2026). In its canonical pathway, CREB is phosphorylated by the CREB-kinase, Akt1, allowing it to bind to DNA in the nucleus and enact transcriptional regulation of genes such as brain-derived neurotrophic factor (Dinevska et al., 2024). One way that DDIT4 inhibits mTOR is via binding and inactivating Akt1 directly (Dennis et al., 2014), suggesting a possible avenue for *Ddit4*-mediated disruptions of CREB activation. Notably, chronic stress exposure, which has been shown to induce elevations in *Ddit4* in the PFC (Ota et al., 2014), causes reductions in the phosphorylation of CREB in the PFC (Kuipers et al., 2003). Intriguingly, a study using an AAV to transfect a dominant-negative inhibitor of CREB in the PFC caused impairments in multiple facets of memory, including temporal order memory (Barker et al., 2020). These results mimicked chronic stress-induced impacts on temporal order memory (Yuen et al., 2012) as well as the memory deficits we observed in our present study. These findings suggest that disruptions in CREB activity in the PFC may play a role in the synaptic and behavioral deficits we observed after *Ddit4* overexpression. More direct studies would be needed to validate these assertions.

As with all scientific experiments, we must acknowledge the limitations of this study. It should be noted that our use of stereotaxic injection of AAVs results in tissue injury and gliosis, which directly affects microglia numbers in our region of interest (Gage et al., 2022). This can contribute to variation in data sampling and results. The AAV construct used here restricted *Ddit4* overexpression to primarily pyramidal neurons in the PFC, so we were unable to examine the role of *Ddit4* in non-neuronal cells. Future studies could use complementary approaches to determine if *Ddit4* overexpression in non-neuronal cells may affect similar synaptic and behavioral responses. Additionally, it would be interesting to examine the effect of *Ddit4* overexpression in subtypes of PFC neurons based on their physiological properties or projection targets. Also, the Thy1-GFP(M) mice exhibited sufficient filling of the dendrites and spine heads but did not adequately fill the spine necks. This limitation was the basis for why we elected not to use any parameters involving dimensions of the spine neck for classifying spines. Our molecular analyses indicated that our AAV construct increased *Ddit4* transcript between 3.68- and 68-fold in the PFC. Prior studies did not measure *Ddit4* transcript but did report a roughly 1.5-fold increase in DDIT4 protein after chronic stress (Ota et al., 2014). The correlation between transcript and protein expression varies across cells (Bauernfeind and Babbitt, 2017), so it is unclear how our *Ddit4* overexpression compares to these other contexts. Further studies are needed to determine how this translates into protein levels and impacts downstream signaling pathways. In addition, we recognize that only male mice were used in these studies. It is well established that sex differences exist in the neurobiology of stress (Woodburn et al., 2021; Bollinger et al., 2023), and future studies should be aimed at determining the role of *Ddit4* overexpression in the female brain.

Collectively, these studies demonstrate that neuronal *Ddit4* overexpression in the PFC of male mice is sufficient to induce deficits in dendritic spine density and associated reductions in temporal order memory. Sequencing analysis revealed that *Ddit4* overexpression caused differential expression of genes involved with synaptic remodeling and mitochondrial function pathways. Contrary to our initial hypothesis, our histological and transcriptomic analyses found no evidence that neuronal *Ddit4* overexpression altered microglia phenotype or function. These results suggest that this “stress-induced” pathway can promote dendritic remodeling in a cell autonomous manner. Given that we observed a predicted “inhibited” state of CREB1 and decreased transcripts associated with mitochondrial function in our RNA sequencing analysis, it is more likely that changes in dendritic spine plasticity following neuronal *Ddit4* overexpression are a result of disruption of the mTOR pathway. Therefore, we conclude that increasing neuronal *Ddit4* expression partially recapitulates stress-induced neurobiological changes in the PFC, including changes to neuronal structure and associated changes in temporal order memory.

## Synthesis

Reviewing Editor: Selva Baltan, Oregon Health &Science University Department of Surgery Decisions are customarily a result of the Reviewing Editor and the peer reviewers coming together and discussing their recommendations until a consensus is reached. When revisions are invited, a fact-based synthesis statement explaining their decision and outlining what is needed to prepare a revision will be listed below. The following reviewer(s) agreed to reveal their identity: NONE. Note: If this manuscript was transferred from JNeurosci and a decision was made to accept the manuscript without peer review, a synthesis may not be available.

Overall the reviewers find the manuscript well written and a valuable contribution but raise several concerns that limit its impact:

1) spine analyses were performed only after forced swim testing (FST), creating a confound because FST effects on spines and Ddit4 are unknown and the timing/rationale for this design is not explained; address why spine density decreased without morphological changes and whether technical issues obscured morphology

2) large animal numbers for behavioral effects suggest subtle

phenotypes;

3) labeling the GO term "Behavior" is uninformative given gene overlap with synaptic categories;

4) the authors should discuss subregional PFC contributions, contextualize the reported ~75× Ddit4 overexpression relative to stress/glucocorticoid-induced changes, and consider neuron-specific (hSyn) versus glial Ddit4 expression and possible glial effects;

5) clarify object engagement measures in the TOR task;

6) integrate RNAseq/PEA/URA findings to explain apparent compensatory increases in dendritic/neuromuscular genes despite spine loss;

7) and better justify or cite mechanisms linking CREB, Akt, and mTOR rather than speculative connections.
